# Neutrophil GM-CSF signaling in inflammatory bowel disease patients is influenced by non-coding genetic variants

**DOI:** 10.1038/s41598-019-45701-2

**Published:** 2019-06-24

**Authors:** Suresh Venkateswaran, Lee A. Denson, Ingrid Jurickova, Anne Dodd, Michael E. Zwick, David J. Cutler, Subra Kugathasan, David T. Okou

**Affiliations:** 1 Department of Pediatrics, Emory University School of Medicine, Atlanta, GA USA; 2 Division of Pediatric Gastroenterology, Hepatology, and Nutrition, Department of Pediatrics, University of Cincinnati College of Medicine and the Cincinnati Children’s Hospital Medical Center, Cincinnati, OH USA; 3 Department of Human Genetics, Emory University, Atlanta, GA USA; 4 Children’s Healthcare of Atlanta, Atlanta, GA USA

**Keywords:** Gastroenterology, Genetics

## Abstract

Neutrophil dysfunction and GM-CSF auto-antibodies are observed in pediatric and adult patients with Crohn’s disease (CD). We associated damaging coding variants with low GM-CSF induced STAT5 stimulation index (GMSI) in pediatric CD patients and implicated variation of neutrophil GM-CSF signaling in cell function and disease complications. Because many CD patients with low GMSI do not carry damaging coding mutations, we sought to test the hypothesis that non-coding variants contribute to this phenotype. We enrolled, performed whole genome sequencing, and measured the GMSI in 77 CD and ulcerative colitis (UC) patients (24 low and 53 normal GMSI). We identified 4 non-coding variants (rs3808851, rs10974787, rs10974788 and rs10974789) in *RCL1* significantly associated with variation of GMSI level (p < 0.011). They were validated in two independent cohorts with: RNAseq data (n = 50) and blood eQTL dataset (n = 31,684). These variants are in LD and affect expression of *JAK2* (p 0.005 to 0.013), *RCL1* (p 8.17E-13 to 2.98E-11) and *AK3* (p 2.00E-68 to 3.03E-55) genes. Additionally, they influence proteins involved in differentiation of gut epithelium, inflammation, and immune system regulation. In summary, our study outlines the contribution of non-coding variants in neutrophil GM-CSF signaling and the potential importance of *RCL1* and *AK3* in CD pathogenesis.

## Introduction

Inflammatory bowel disease (IBD) is comprised of Crohn’s Disease (CD) and ulcerative colitis (UC). Genomic and functional studies of CD suggest that Granulocyte-Macrophage Colony Stimulating Factor (GM-CSF) plays a crucial role in the interactions between host and microbe^1–3^. An increase in GM-CSF auto-antibodies (GMAb) that suppress GM-CSF:STAT5 signaling has been associated with neutrophil dysfunction in pediatric and adult CD patients^4,5^. Recently, using peripheral blood samples of 543 pediatric IBD patients and whole exome sequencing (WES), we tested for association between coding variants and the GM-CSF:STAT5 signaling pathway in neutrophils, and disease complications^6^. We identified coding variants in highly conserved sites in the GM-CSF signaling genes *CSF2RA*, *CSF2RB*, *JAK2*, *STAT5A* and *STAT5B*^6^. In particular, we observed that patients with low neutrophil GM-CSF induced STAT5 stimulation index (low GMSI) carry more damaging missense mutations and have a reduced ability of neutrophils to kill Staph Aureus. Additionally, stricturing behavior is increased in CD patients who have both low GMSI and elevated GMAb^7^. However, we noted that damaging missense mutations are not found in most of the CD patients who have low GMSI, which suggests the hypothesis that the low GMSI trait could be influenced by non-coding variants that regulate GM-CSF signaling. Many genotypes associated with diseases are located in regulatory regions and are more likely to be expression quantitative trait loci (eQTLs)^8,9^. These non-coding and regulatory variants are best ascertained through comprehensive genomic dissection via whole genome sequencing (WGS). Also, quantitative variation in transcription frequently leads to protein variation^10^, which lead to phenotypic traits. Therefore, evaluation of transcripts is important and is best done through RNA sequencing (RNAseq). Unlike genome-wide association study (GWAS) of disease or clinical phenotypes^11^, eQTL analysis can provide significant results with few samples^12^. Understanding the drivers and modifiers of GM-CSF signaling in CD patients who have low GMSI but do not carry damaging missense mutations could offer further insight into CD pathogenesis. Therefore, we asked whether CD patients selected on the basis of their low neutrophil GMSI level would carry non-coding regulatory variants for gene expression in neutrophils, that affect GM-CSF signaling. To test our hypothesis that non-coding variants are implicated in GMSI levels, we recruited CD and UC patients on the basis on their GMSI level and performed WGS to comprehensive variant identification genetic variants. We also attempted to replicate variants previously associated with neutrophil GMSI signaling in pediatric CD^6^.

## Results

Using WES in pediatric CD, we previously identified potentially damaging rare missense coding mutations in GM-CSF signaling genes^6^ and we reported that the protein variant p.Ala17Gly (MAF = 9%) of GM-CSF Receptor Alpha Chain (CSF2RA) was associated with low GMSI in CD patients^6^. To identify non-coding regulatory variants for gene expression in neutrophils that affect GM-CSF signaling and to replicate previous findings for association between GM-CSF:STAT5 signaling pathway in neutrophils, and coding variants, we recruited 77 patients with known GMSI level comprised of 39 CD, 36 UC, 1 inflammatory bowel disease-undetermined (IBD-U) and 1 control (Supplementary Fig. 1 and Supplementary Table 1) and we performed WGS on them. These included 39 CD patients and 36 UC patients. Following mapping, variant calling, annotation, and stringent filtering, we replicated previously reported variants p.Pro603Thr and p.Pro696Ser of *CSF2RB*^6^ in 10% (n = 8) and 5% (n = 4) of patients respectively. Of all patients carrying the replicated *CSF2RB* variants, only 2.5% had low GMSI (≤25%) while others had high GMSI (>25%), consistent with our recent report. We also identified 5 potentially damaging coding variants (with Combined Annotation-Dependent Depletion (CADD) score from 11 to 35) not previously identified by WES, which were validated using the Sanger sequencing method (supplementary Table 2).

**Figure 1:**
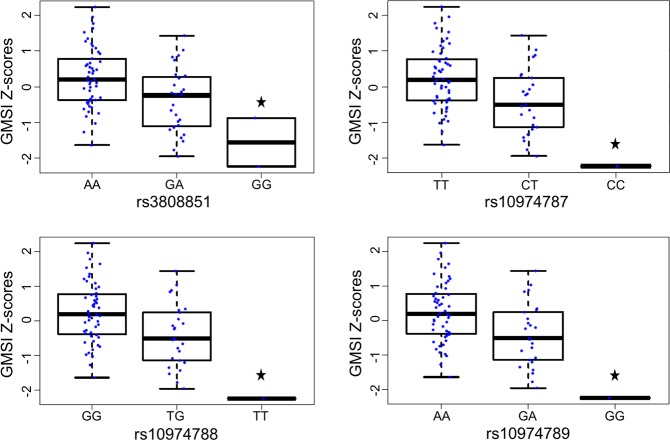
Boxplot of four *RCL1* variants associated with GM-CSF induced STAT5 stimulation index (GMSI). *Indicates the homozygous genotypes for each variant significantly associated with reduced GMSI level.

**Figure 2:**
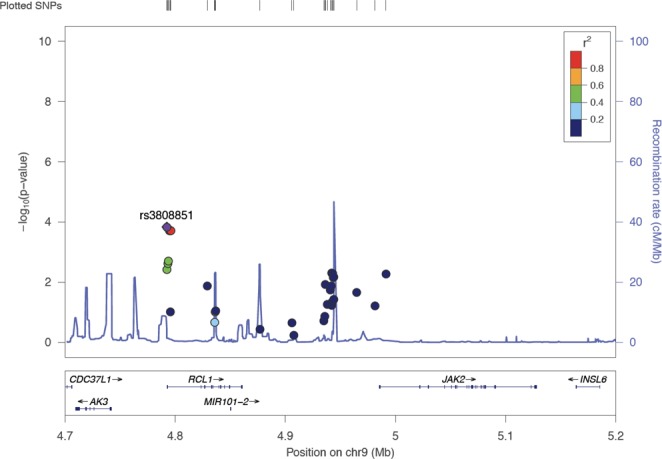
LocusZoom plot of SNPs by chromosome position against −log10 P value for their genetic associations with low GMSI. The top SNP (rs3808851) is highlighted in purple. The surrounding SNPs, shown within 100 to 500 kb of the top SNP are color-coded to reflect their linkage disequilibrium in r^2^ (see inset key) with the top SNP. The estimated recombination rates are plotted in pale blue to reflect local LD structure on secondary y-axis.

To understand the drivers and modifiers of low GMSI, we assessed genotypes within 1 Mb of the GM-CSFR Alpha and Beta chain genes transcription start site (TSS) (*CSF2RA*, *CSF2RB*, *JAK2*, *STAT5A and STAT5B*) for association with low GMSI. This resulted in 8,923 total variants, of which 231 significant variants were retained by testing for chi-square p-value < 0.05 between low GMSI (≤25%) and high GMSI (>25%). Next, we asked which genotype groups are associated GMSI level. Out of the 231 variants retained, genotypes for only 4 single nucleotide polymorphisms (SNPs) rs3808851, rs10974787, rs10974788 and rs10974789 were significantly associated with variation of GMSI level (p < 0.011). In particular, the homozygous genotypes rs3808851-G, rs10974787-C, rs10974788-T, rs10974789-G were associated with reduced GMSI level (p < 0.011) in neutrophils (Fig. 1). Those 4 SNPS map to the non-coding region of the gene *RCL1*. A locusZoom analysis showed that all of the 4 non-coding *RCL1* SNPs are in perfect linkage disequilibrium (LD) with each other (r^2^ ≥ 0.8) (Fig. 2). The annotation of those non-coding *RCL1* variants within the LD block showed that rs10974787, rs10974788 and rs10974789 are intronic while rs3808851 is located ~500 bp upstream of *RCL1*. With the exception of rs10974788, all other 3 SNPs affect the regulation of many transcription factor binding activities (as reported by SNP2TFBS^13^). Additionally, rs3808851 is located in a binding site for the nuclear factor-κB (NFKB) and the KRAB-associated protein-1 (KAP1) proteins (Table 1). The annotation also reports the AK3 protein as an eQTL hit for SNPs rs3808851, rs10974787 and rs10974788.

**Table 1:** Annotation of SNPs associated with low GMSI (queried SNP rs3808851 and LD variants with r^2^ ≥ 0.8).

chr	pos (hg38)	LD (r²)	LD (D’)	variant	Ref	Alt	Frequency		Promoter histone marks	DNAse	Proteins bound	Motifs changed	GRASP QTL hits	Selected eQTL hits	GENCODE genes	dbSNP func annot
							AFR	EUR								
9	4792371	1	1	rs3808851	A	G	0.24	0.2	24 tissues	37 tissues	NFKB, KAP1	ERalpha-a, GR	*AK3*	*AK3*	497 bp 5′ of *RCL1*	—
9	4794758	0.8	0.95	rs10974787	T	C	0.1	0.19	—		—	Arid5a, HNF1, Lhx3, Sox	—	*AK3*	*RCL1*	intronic
9	4795595	0.8	0.95	rs10974788	G	T	0.07	0.19	—	IPSC	—	—	AK3	*AK3*	*RCL1*	intronic
9	4796032	0.8	0.95	rs10974789	A	G	0.07	0.19	—	Blood	—	GATA, TAL1	—	—	*RCL1*	intronic

**Figure 3:**
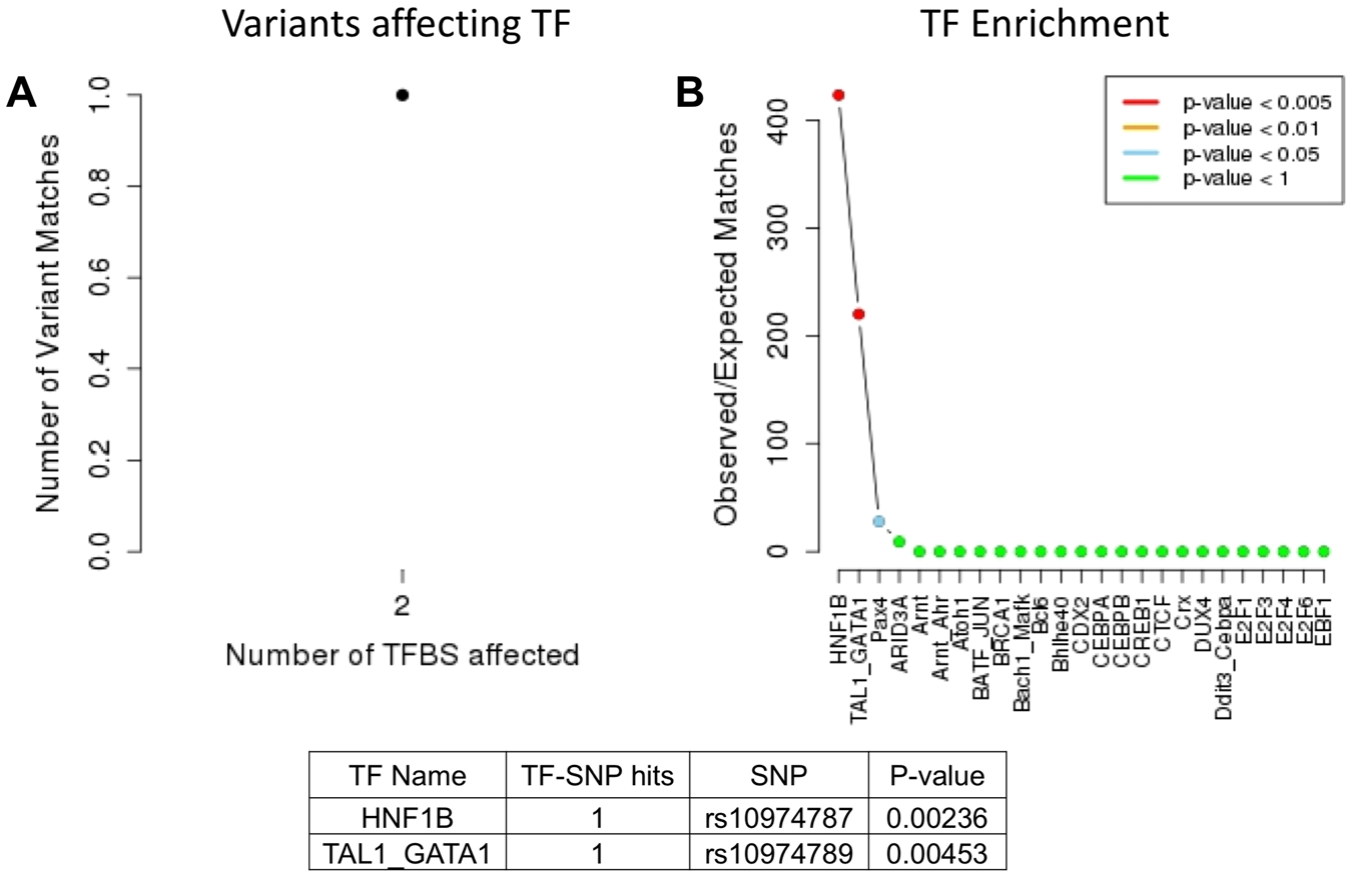
(**A**) Two transcription factor binding sites (TFBS) are affected by 1 variant identified in patients with low GMSI. (**B**) The two TFBS are significantly associated with the transcription factors HNF1B and TAL1_GATA1.

To determine which transcription factors were affected by the SNPs most significantly associated with the variation of GMSI level, we performed an *in-silico* analysis using the Web interface SNP2TFBS^13^. Our analysis showed that HNF1B and TAL1/GATA1 were the only transcription factors (TFs) enriched and specifically linked to rs10974787 and rs10974789 respectively, in association with the reduction of GMSI level (Fig. 3A) at significant p value of 0.00236 and 0.00453 respectively (Fig. 3B), which implicates a role in GMSI signaling. The enrichment of these TFs was not observed in our RNAseq analysis of an independent IBD cohort with low or normal GMSI.

To validate our finding that 4 SNPs belonging to *RCL1* are associated with reduced GMSI level, an eQTL analysis (with and without covariates) was performed on an independent IBD cohort (n = 50) with previously available RNASeq data. Results showed that statistically, rs3808851 has no direct significant influence on the expression of any of the tested genes (*CSF2RA*, *CSF2RB*, *JAK2 STAT5A and STAT5B*) implicated in GM-CSF signaling. However, a t-test showed that the homozygous genotype GG of rs3808851 showed significant differences in gene expression level for *JAK2* (p = 0.005 compared to AG and p = 0.0133 compared to AA) and for *AK3* (p = 0.031 compared to AG). Our eQTL analysis and t-test results are reported in Supplementary Table 3.

We next sought to replicate our findings in a much larger cohort with existing blood eQTL data from Europeans. Results showed that the four variants strongly associate with 2 nearby genes; *AK3* with p < 3.03E-55 to 1.49E-66 (downstream: distance between −65KB to −70KB to the center of the gene) and *RCL1* with p < 2.98E-11 to 9.17E-13 (upstream: distance between 30KB to 34KB to the center of the gene). None of the variants were associated with *JAK2* using this larger cohort blood eQTL dataset. Results are reported in Supplementary Table 4.

## Discussion

GM-CSF plays a crucial role in the priming of neutrophil antimicrobial functions. In pediatric CD, we have established an association between the GM-CSF:STAT5 signaling pathway in neutrophils, disease complications, and coding variants^6^. We reported that patients with low GMSI have an increased frequency of damaging missense mutations in the GM-CSF Receptor Alpha Chain genes. However, damaging missense mutations are not found in most CD patients who have low GMSI. Many genetic studies of complex diseases report associations that localize to noncoding or incompletely characterized regulatory regions, which likely represent expression quantitative trait loci (eQTLs)^8,9^. Unlike GWAS of disease or clinical phenotypes^11^, eQTL analysis can provide significant results with as few as 100 samples^12^. We, therefore, recruited CD and UC patients on the basis on their GMSI results and performed WGS to not only replicate previously identified coding variants but also to identify non-coding variants implicated in the regulation of neutrophil-intrinsic GMSI.

From our WGS analysis, we replicated two coding variants (p.Pro603Thr and p.Pro696Ser) of the gene *CSF2RB*, previously identified by WES. Theses replicated coding variants were 2 of only 3 (66%) found at 3% or higher frequency in the WES cohort. We did not replicate p.Ala17Gly (9% frequency), which supports our previous report that only a fraction of the variation in GMSI level was associated with the p.Ala17Gly mutation in the CSF2RA protein^6^. The fact that none of the previously identified coding variants with frequency <3% were replicated is attributed to our small sample size.

In *RCL1*, we identified four (4) SNPs that are in strong LD, for which homozygous genotypes are directly associated with reduced GMSI level (p < 0.011) in neutrophils. The *RCL1* gene codes for the RNA 3′-terminal phosphate cyclase-like protein, an endonuclease^14^ without cyclase activity, implicated in ribosome biogenesis (plays a role in 40S-ribosomal-subunit biogenesis in early pre-rRNA processing steps at sites A0, A1 and A2 that are required for proper maturation of the 18 S RNA). From the iPTMnet database, humans have 2 isoforms of RCL1 with substrate role and containing 19 post-translational modification (PTM) sites, of which 15 phosphorylation, 2 methylation and 2 ubiquitination sites^15^. While PTMs are known to influence proteins activities^16,17^, we have no evidence that the 4 non-coding *RCL1* SNPs identified in this study affect PTM of RCL1. In yeast, Rcl1- mutant strains showed decreased levels of 40 S ribosomal subunits, resulting in decreases of 20 S pre-rRNA and 18 S rRNA levels^18^. The *RCL1* gene has been nominally (p < 0.05) associated with IBD susceptibility by large scale GWAS studies in European ancestry participants, and is known to interact with *JAK2*^19,20^. Other studies have also reported eQTL effects between *RCL1* and *AK3*^21,22^. Additionally, SNP rs10974788 of *RCL1* was reported to associate with CD only in the Ashkenazi Jewish population^23^. Studies have also outlined a role for gut microbiome in initiating and driving IBD, in support of a host-microbiome interaction^24–26^. In term of host genetic susceptibility, it is likely that the 4 non-coding *RCL1* SNPs influence the gut microbiome. However, our study design did not explore the association patterns between the identified four non-coding *RCL1* SNPs and the commensal microbiome.

Through validation, this study not only lends further and stronger support to the *RCL1*-*JAK2* and *RCL1*-*AK3* interactions but also shows that low-GMSI trait could be attributed to non-coding variants that regulate GM-CSF signaling. In our WGS cohort, genotype GG of the non-coding SNP rs3808851 affected the expression of both *JAK2* and *AK3*.

Of the identified four SNPs associated with reduced GMSI level, three (rs3808851, rs10974787 and rs10974789) affect regulation of various transcription factors (TF) that are not directly implicated in GM-CSF signaling (Table 1). In particular, intronic SNPs rs10974787 and rs10974789 within the LD block appear to have the greatest influence on the regulation of TF, as they associate with enrichment of *HNF1B* and *TAL1/GATA1* respectively. In fact, both *HNF1B* and *TAL1/GATA1* represent regulatory motifs reported to be affected by rs10974787 and rs10974789^27^. The HNF1B protein is a transcription factor found in many tissues, including the intestines. It can act either as a homodimer or as a heterodimer with HNF1A, another closely related protein. Along with its partners, HNF1B plays a role both in cell fate decision and terminal differentiation in the gut epithelium, by directly controlling the expression of *SLC26A3* in the intestinal epithelium^28^. HNF1B has not been implicated in IBD. TAL1 and GATA1 are well-established blood cell regulators whereby TAL1 plays multiple key roles in hematopoiesis and is needed for differentiation of erythroid progenitor cells into maturing erythroblasts^29^. The co-binding of GATA1 with TAL1 into a complex is strongly associated with gene expression induction, while GATA1 without TAL1 represses gene expression^30^. Both TAL1 and GATA1 have also not been implicated in IBD.

SNP rs3808851, which is located ~500 bp upstream from *RCL1*, is known to bind nuclear factor-κB (NFKB) and KRAB-associated protein-1 (KAP1) proteins in ChIP-Seq experiments^31^. *NFKB* is known to correlate with CD phenotypes and contribute significantly to the chronic inflammation underlying IBD^32,33^. KAP1 (aka TRIM28 or TIF1β) is known for its role in diverse cellular processes including epigenetic regulation of cell function. However, KAP1 has recently been reported in the mouse to act as a transcription factor partner of FOXP3 that promotes regulatory T cells (Treg) gene expression and to play a critical role in cell-cycle progression and Tregs proliferation^34^. SNP rs3808851 is also known to affect regulation of estrogen receptor alpha (ERα, aka ESR1) and glucocorticoid receptor (GR, aka NR3C1). There is evidence in mouse models of IBD, that signaling through ERα results in pro-inflammatory response sand that loss of ERα decreases disease severity^35^. GR is known to mediate glucocorticoid action and play an important role in inflammatory responses. In patients with IBD, the expression of GR is altered, which affects mucosal repair and intestinal barrier function^36^. Previous studies of genotype to phenotype correlation have also associated rs3808851 with type 2 diabetes^37,38^.

We have shown that in IBD patients stratified by neutrophil intrinsic GM-CSF signaling, low GMSI is driven by non-coding variants that significantly affect the expression of *RCL1*, *JAK2* and *AK3* genes and the binding of transcription factors that play a role in differentiation of gut epithelium, inflammation and regulation of the immune system. This study underscores the importance of eQTL studies in revealing the contribution of the non-coding variant to disease and in identifying genes which play indirect roles and should be considered when investigating the pathogenesis of IBD.

## Methods

### Population

This study included 77 pediatric IBD patients (24 low and 53 normal GMSI) enrolled in the Cincinnati Children’s Hospital sponsored RISK cohort study with ancillary genomic and functional studies supported by the National Institutes of Health (NIH). These samples belong to a cohort of 129 consecutive IBD patients for whom neutrophil functional studies had been completed, and genomic DNA was available for genotyping. The clinical and demographic features of the 77 subjects are provided in Supplementary Table 1. Patients were comprised of CD (n = 39), UC (n = 36) inflammatory bowel disease-undetermined (IBD-U; n = 1) and control (n = 1).

Informed consent was obtained from each participant or from the legal guardians of minors. The Institutional Review Boards of Emory University and the Cincinnati Children’s Hospital approved the study protocol. All experiments and methods were carried out in accordance with the relevant guidelines and regulations applied at Cincinnati Children’s Hospital and of Emory University.

### Whole genome sequencing and variants analysis

Sequencing was done at the HudsonAlpha Institute for Biotechnology (Huntsville, AL). Briefly, libraries for DNA extracted from whole blood of selected 77 IBD patients were prepared according to the manufacturer’s instructions, and sequenced on the Illumina HiSeq platform. Mapping of the raw sequence reads (against the human genome reference sequence, build hg38) and calling of variants were done using PEmapper and PECaller respectively^39^. The functional annotation of these variants was performed with Bystro^40^ which also reported whether any variants were present in dbSNP or were deleterious based on a Combined Annotation-Dependent Depletion (CADD) scores of 10 or higher^41^. The Genome Aggregation Database (gnomAD) reference dataset was used for estimation of allele frequency^42^.

### Neutrophil GM-CSF stimulation index

The assay was performed using peripheral blood samples obtained from 76 pediatric IBD patients and 1 healthy control. The peripheral blood was lysed, and the cells were washed in Dulbecco’s phosphate-buffered saline (DPBS). Washed neutrophils were used to remove circulating GM-CSF auto-antibodies. Then cells were resuspended in RPMI medium +/− stimulation with 10 ng/ml of GM-CSF for 20 minutes at 37 °C. The cells were fixed overnight at 4 °C with 1% paraformaldehyde. The next day, the cells were permeabilized with cold 100% methanol (stored at −20 °C). The cells were then washed and stained for intracellular STAT5 with Anti-pSTAT5 (pY694) antibody (BD Bioscience, San Jose, CA). A minimum of 10^4^ cells were acquired on flow cytometer (FACS-Calibur, Becton Dickinson) and analyzed with the instrument software CellQuest and DeNovo (Supplementary Fig. 1). The GM-CSF Stimulation Index (GMSI) was calculated from mean fluorescence intensity (MFI) from granulocyte gate as follows GMSI = (GM-CSF stimulated MFI - unstimulated MFI)/unstimulated MFI × 100. The GMSI was used to define GM-CSF signaling in neutrophils. We characterized a low GMSI (GMSI Lo) as ≤ 25%, where the 20^th^ percentile falls for all tested controls. The GMSI values were converted into a standard normal distribution with mean 0 and variance 1 (Z-score), using in-house R script.

### Filtering the GM-CSF associated variants

Using whole exome sequencing (WES) of 543 pediatric IBD patients, we recently identified potentially damaging coding rare variants in the GM-CSF signaling genes *CSF2RA*, *CSF2RB*, *JAK2 STAT5A and STAT5B*^6^. Thus, we selected all the variants located within ± 500 Kb (Total 1 Mb) region from the gene’s transcription starts site (TSS), which were filtered using plink1.09 software^43^ and by testing for chi-square p-value < 0.05 between two groups: GMSI ≤ 25 and GMSI > 25 using the R package.

### Association study of variants on GM-CSF signaling

We used genome-wide efficient mixed-model association algorithm, GEMMA^44^, to assess the effect of genetic variants on the intrinsic level of GM-CSF signaling. GEMMA allows the user to adjust for population structure and relatedness among individuals as a random effect through a genetic relationship matrix (GRM) using the variants retained after quality control steps. In addition to the GRM, the data were adjusted for age, gender, race and disease status. Finally, in order to detect effective variants, the association was performed between normalized GMSI and 231 filtered variants located within 1 Mb regions of GM-CSF signaling associated genes transcription start site (TSS). A significant association between GMSI level and genotypes was detected by filtering the results with adjusted FDR p-value < 0.05. HaploReg^45^ was used to annotate and to prioritize SNP within LD block, and SNP2TFBS^13^ was used to determine the transcription factor (TF) binding sites affected by selected SNPs and to determine which TF were enriched in association with low GMSI.

### Genotypes and correlation with gene expression- eQTL analysis

To validate these four SNPs in LD, we performed an eQTL analysis using an internal independent cohort of 50 IBD patients with pre-existing RNASeq data to determine if variants associated with GMSI level also influence gene expression. We performed Taqman genotyping of SNP rs3808851 (one of two SNPs with available Taqman assay) on DNA of 50 IBD subjects (CD = 36; UC = 9 and control = 5) with pre-existing RNAseq data. We tested for significant eQTL association of rs3808851 on the expression of *RCL1*, on the expression of GM-CSF signaling genes (*CSF2RA*, *CSF2RB*, *JAK2 STAT5A*, *and STAT5B*) and on the expression of transcription factors (TAL1, AK3, HNF1B). Each gene was tested in linear regression (lm) with normalized Z-score from read counts against the rs3808851 (AA = 32; AG = 13; and GG = 4), where age, sex, gender, disease types were added as covariates. We also performed a simple t-test to determine whether the expression of a gene is statistically significant within its corresponding genotype groups without any genetic influences. Statistical analysis was done using the R package and the p-value** < **0.05 were considered statistically significant in both tests.

### Validation of GM-SCF associated variants in larger blood eQTL study from European cohort

Owing to the small sample size of our internal validation cohort that provided low power to our eQTL analysis, we used publically available eQTL from blood performed by Võsa *et al*.^46^ through the eQTLGen Consortium (www.eqtlgen.org) consisting of 31,684 European individuals. In this larger cohort, we tested the *cis-* and *trans* effect of the four variants associated with reduced GMSI level, on gene expression of *RCL1*, GM-CSF signaling genes (*CSF2RA*, *CSF2RB*, *JAK2 STAT5A*, *and STAT5B*) and transcription factors (TAL1, AK3, HNF1B) to further validate our finding.

## Supplementary information

Supplementary information

## Data Availability

The datasets generated during and/or analyzed during the current study are available from the corresponding author upon request.

## References

[1] Jostins L (2012). Host-microbe interactions have shaped the genetic architecture of inflammatory bowel disease. Nature.

[2] Chuang LS (2016). A Frameshift in CSF2RB Predominant Among Ashkenazi Jews Increases Risk for Crohn’s Disease and Reduces Monocyte Signaling via GM-CSF. Gastroenterology.

[3] Levine AP (2016). Genetic Complexity of Crohn’s Disease in Two Large Ashkenazi Jewish Families. Gastroenterology.

[4] Gathungu, G. *et al*. Granulocyte-Macrophage Colony-Stimulating factor auto-antibodies: a marker of aggressive crohn’s disease. *Inflamm Bowel Dis***in press** (2013). 10.1097/MIB.0b013e318281f506 PMC3707315 23749272

[5] Han X (2009). Granulocyte-macrophage colony-stimulating factor autoantibodies in murine ileitis and progressive ileal Crohn’s disease. Gastroenterology.

[6] Denson LA (2019). Genetic and Transcriptomic Variation Linked to Neutrophil Granulocyte-Macrophage Colony-Stimulating Factor Signaling in Pediatric Crohn’s Disease. Inflamm Bowel Dis.

[7] Jurickova I (2013). Paediatric Crohn disease patients with stricturing behaviour exhibit ileal granulocyte-macrophage colony-stimulating factor (GM-CSF) autoantibody production and reduced neutrophil bacterial killing and GM-CSF bioactivity. Clin Exp Immunol.

[8] Gusev A (2014). Partitioning heritability of regulatory and cell-type-specific variants across 11 common diseases. Am J Hum Genet.

[9] Nicolae DL (2010). Trait-associated SNPs are more likely to be eQTLs: annotation to enhance discovery from GWAS. PLoS Genet.

[10] Dimas AS (2009). Common regulatory variation impacts gene expression in a cell type-dependent manner. Science.

[11] Visscher P, Brown M, McCarthy M, Yang J (2012). Five years of GWAS discovery. Am J Hum Genet.

[12] Gibson G, Powell JE, Marigorta UM (2015). Expression quantitative trait locus analysis for translational medicine. Genome Med.

[13] Kumar S, Ambrosini G, Bucher P (2017). SNP2TFBS - a database of regulatory SNPs affecting predicted transcription factor binding site affinity. Nucleic Acids Res.

[14] Gaudet P, Livstone MS, Lewis SE, Thomas PD (2011). Phylogenetic-based propagation of functional annotations within the Gene Ontology consortium. Brief Bioinform.

[15] Huang H (2018). iPTMnet: an integrated resource for protein post-translational modification network discovery. Nucleic Acids Res.

[16] Christiansen MN (2014). Cell surface protein glycosylation in cancer. Proteomics.

[17] Snider NT, Omary MB (2014). Post-translational modifications of intermediate filament proteins: mechanisms and functions. Nat Rev Mol Cell Biol.

[18] Billy E, Wegierski T, Nasr F, Filipowicz W (2000). Rcl1p, the yeast protein similar to the RNA 3′-phosphate cyclase, associates with U3 snoRNP and is required for 18S rRNA biogenesis. EMBO J.

[19] Barrett JC (2008). Genome-wide association defines more than 30 distinct susceptibility loci for Crohn’s disease. Nat Genet.

[20] Liu JZ (2015). Association analyses identify 38 susceptibility loci for inflammatory bowel disease and highlight shared genetic risk across populations. Nat Genet.

[21] Fehrmann RS (2011). Trans-eQTLs reveal that independent genetic variants associated with a complex phenotype converge on intermediate genes, with a major role for the HLA. PLoS Genet.

[22] Westra HJ (2013). Systematic identification of trans eQTLs as putative drivers of known disease associations. Nat Genet.

[23] Kenny EE (2012). A genome-wide scan of Ashkenazi Jewish Crohn’s disease suggests novel susceptibility loci. PLoS Genet.

[24] Turnbaugh PJ (2007). The human microbiome project. Nature.

[25] Peterson DA, Frank DN, Pace NR, Gordon JI (2008). Metagenomic approaches for defining the pathogenesis of inflammatory bowel diseases. Cell Host Microbe.

[26] Aschard H (2019). Genetic effects on the commensal microbiota in inflammatory bowel disease patients. PLoS Genet.

[27] Kheradpour P, Kellis M (2014). Systematic discovery and characterization of regulatory motifs in ENCODE TF binding experiments. Nucleic Acids Res.

[28] D’Angelo A (2010). Hepatocyte nuclear factor 1alpha and beta control terminal differentiation and cell fate commitment in the gut epithelium. Development.

[29] Porcher C (1996). The T cell leukemia oncoprotein SCL/tal-1 is essential for development of all hematopoietic lineages. Cell.

[30] Cheng Y (2009). Erythroid GATA1 function revealed by genome-wide analysis of transcription factor occupancy, histone modifications, and mRNA expression. Genome Res.

[31] Dunham I (2012). An integrated encyclopedia of DNA elements in the human genome. Nature.

[32] McDaniel DK, Eden K, Ringel VM, Allen IC (2016). Emerging Roles for Noncanonical NF-kappaB Signaling in the Modulation of Inflammatory Bowel Disease Pathobiology. Inflamm Bowel Dis.

[33] Han YM (2017). NF-kappa B activation correlates with disease phenotype in Crohn’s disease. PLoS One.

[34] Tanaka S (2018). KAP1 Regulates Regulatory T Cell Function and Proliferation in Both Foxp3-Dependent and -Independent Manners. Cell Rep.

[35] Cook LC (2014). The role of estrogen signaling in a mouse model of inflammatory bowel disease: a Helicobacter hepaticus model. PLoS One.

[36] Nagy Z (2016). Overexpression of GRss in colonic mucosal cell line partly reflects altered gene expression in colonic mucosa of patients with inflammatory bowel disease. J Steroid Biochem Mol Biol.

[37] Leslie R, O’Donnell CJ, Johnson AD (2014). GRASP: analysis of genotype-phenotype results from 1390 genome-wide association studies and corresponding open access database. Bioinformatics.

[38] Dupuis J (2010). New genetic loci implicated in fasting glucose homeostasis and their impact on type 2 diabetes risk. Nat Genet.

[39] Johnston, H. R. *et al*. PEMapper and PECaller provide a simplified approach to whole-genome sequencing. *Proc Natl Acad Sci USA* (2017). 10.1073/pnas.1618065114 PMC5347547 28223510

[40] Kotlar AV, Trevino CE, Zwick ME, Cutler DJ, Wingo TS (2018). Bystro: rapid online variant annotation and natural-language filtering at whole-genome scale. Genome Biol.

[41] Kircher M (2014). A general framework for estimating the relative pathogenicity of human genetic variants. Nat Genet.

[42] Lek M (2016). Analysis of protein-coding genetic variation in 60,706 humans. Nature.

[43] Purcell S (2007). PLINK: a tool set for whole-genome association and population-based linkage analyses. Am J Hum Genet.

[44] Zhou X, Stephens M (2012). Genome-wide efficient mixed-model analysis for association studies. Nat Genet.

[45] Ward LD, Kellis M (2012). HaploReg: a resource for exploring chromatin states, conservation, and regulatory motif alterations within sets of genetically linked variants. Nucleic Acids Res.

[46] Võsa, U. *et al*. Unraveling the polygenic architecture of complex traits using blood eQTL meta-analysis. *bioRxiv*, 447367 (2018).

