# Supplementary material for: Neutrophil GM-CSF signaling in inflammatory bowel disease patients is influenced by non-coding genetic variants
Source: Sci Rep. 2019 Jun 24;9:9168. doi: 10.1038/s41598-019-45701-2 (PMC6591305; doi:10.1038/s41598-019-45701-2)
Supplement: Supplementary file 1 — Supplementary information [file 41598_2019_45701_MOESM1_ESM.pdf]

Neutrophil GM-CSF signaling in inflammatory bowel disease patients is influenced by non-coding genetic variants

Suresh Venkateswaran[1], Lee A. Denson[2], Ingrid Jurickova[2], Anne Dodd[1], Michael E. Zwick[3], David J. Cutler[3], Subra Kugathasan[1,4] and David T. Okou[1*]

1. Department of Pediatrics, Emory University School of Medicine, Atlanta, GA, USA
2. Division of Pediatric Gastroenterology, Hepatology, and Nutrition, Department of Pediatrics, University of Cincinnati College of Medicine and the Cincinnati Children's Hospital Medical Center, Cincinnati, OH, USA
3. Department of Human Genetics, Emory University, Atlanta, GA, USA
4. Children's Healthcare of Atlanta, Atlanta, GA, USA

SUPPLEMENTARY INFORMATION

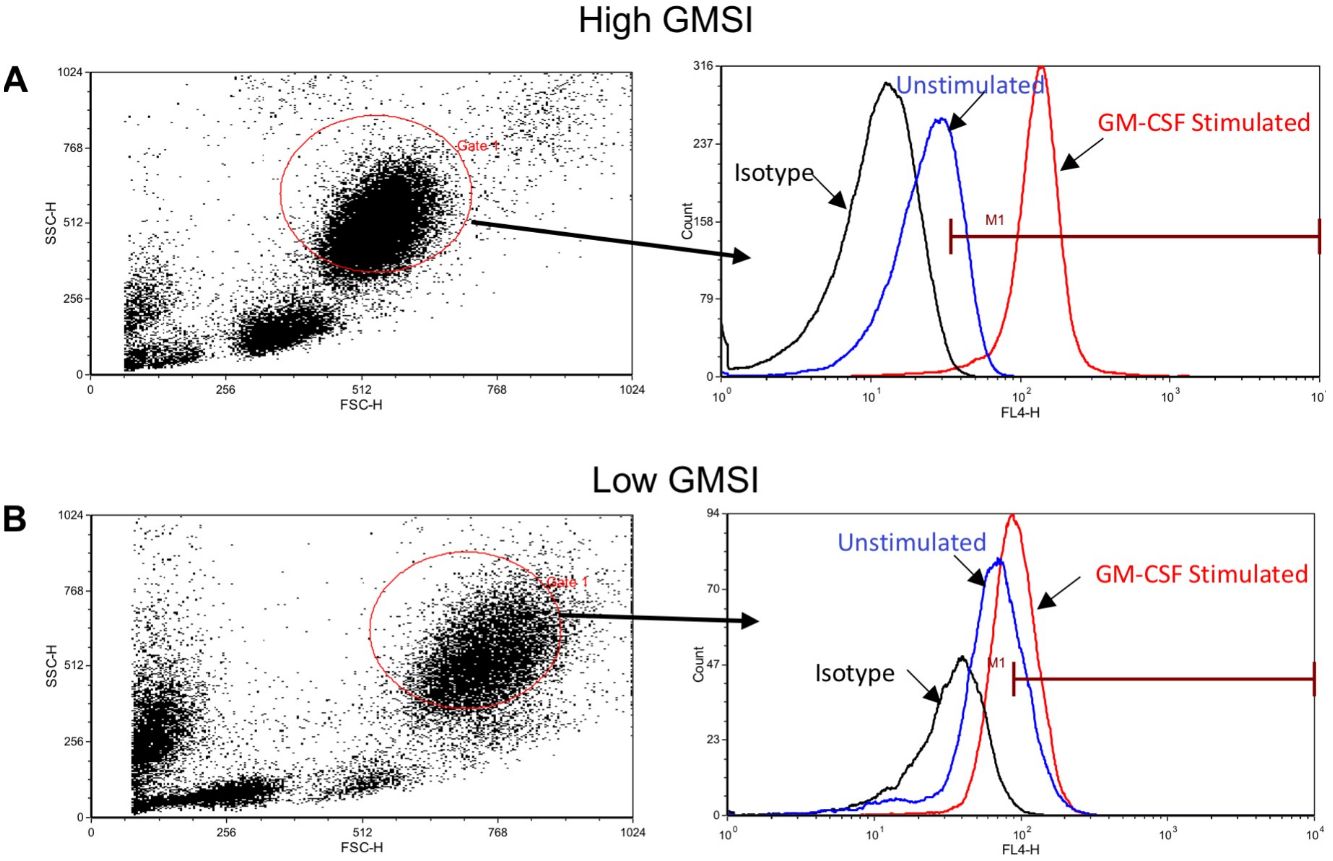

Supplementary Figure 1. Intracellular flow cytometry for pSTAT5. Peripheral blood neutrophils were lysed, washed and +/-stimulated with GM-CSF. (A) IBD patient with high GMSI and (B) IBD patient with low GMSI in neutrophils.

	**CD (**n=39**)**	**UC (**n=36)	**IBD-U (n=1)**	**Control (n=1)**
Mean age (SD)	10.2 (4.5)	9.7 (4.47)	17 (NA)	17 (NA)
Female (%)	15 (38.47)	22 (61.11)	1 (100)	0
Male (%)	24 (61.53)	14 (38.89	0	1 (100)
African American (%)	4 (10)	3 (8.33)	0	0
Caucasian (%)	35 (90)	32 (88.89)	1 (100)	0
Bi-Racial (%)	0	1 (2.78)	0	1 (100)
GMSI Lo - ≤ 25% (%)	11 (28.20)	12 (33.33)	1 (100)	0
GMSI Hi - > 25% (%)	28 (71.80)	24 (66.67)	0	1 (100)

Supplementary Table 1. Study cohort clinical and demographic characteristics

Gene	Chr	Start	Transcript change	protein change	dbSNP ID	MAF in cohort	MAF (population)		
							AFR	EUR	
CSF2RB	22	36937615	c.C1807A	p.Pro603Thr	rs1801122	0.05128	0.0054	0.0449	replicated
CSF2RB	22	36937894	c.C2086T	p.Pro696Ser	rs16997517	0.02564	0.1168	0.0197	replicated
CSF2RB	**22**	**36930401**	**c.G745C**	**p.Glu249Gln**	**rs16845**	**0.07051**	**0.2215**	**0.0549**	**WGS only**
JAK2	**9**	**5065003**	**c.C1177G**	**p.Leu393Val**	**rs2230723**	**0.01923**	**0.0272**	**0.005**	**WGS only**
STAT5A	**17**	**42301451**	**c.G1166A**	**p.Arg389His**	**rs2230134**	**0.00641**	**0.0103**	**7.17E-05**	**WGS only**
STAT5B	**17**	**42207663**	**c.G1972A**	**p.Asp658Asn**	**rs148568485**	**0.00641**	**0**	**8.95E-06**	**WGS only**
STAT5B	**17**	**42224826**	**c.C328T**	**p.Arg110Cys**	**rs775502922**	**0.00641**	**0**	**4.48E-05**	**WGS only**

Supplementary Table 2. GM-CSF Alpha and Beta chain gene variants identified by WGS (this study). Variants in bold were identified in WGS only and validated by Sanger sequencing.

Genes	lm_with covarites	lm_without covariates	t-test AA-AG	t-test AG-GG	t-test AA-GG
JAK2	0.104	0.110	0.204	**0.005**	**0.013**
AK3	0.342	0.328	0.327	**0.031**	0.101
STAT5A	0.467	0.449	0.912	0.300	0.306
TAL1	0.387	0.409	0.181	0.314	0.062
STAT5B	0.871	0.869	0.745	0.544	0.662
HNF1B	0.603	0.599	0.569	0.558	0.651
CSF2RA_X	0.933	0.933	0.994	0.674	0.650
CSF2RB	0.960	0.959	0.936	0.788	0.813

Supplementary Table 3. eQTL and t-test analysis with rs3808851 and GM-CSF signaling genes in an independent cohort (n=50). Genes in bold have their expression significantly affected by genotypes from t-test.

						AK3 (ENSG00000147853) 9:4726599 - downstream			*RCL1* (ENSG00000120158) 9:4826966 - upstream			*JAK2 (ENSG00000096968) 9:5056608*		
SNP	Chr	SNP position	A1	A2	Freq	b	SE	p	b	SE	p	b	SE	p
rs3808851	9	4792371	G	A	0.235	-0.164	0.0105	3.03E-55	-0.070	0.0105	2.98E-11	NA	NA	0.76600847
rs10974787	9	4794758	C	T	0.173	-0.205	0.0117	2.00E-68	-0.085	0.0118	8.17E-13	NA	NA	0.12851098
rs10974788	9	4795595	T	G	0.166	-0.207	0.0119	1.72E-67	-0.086	0.0120	9.16E-13	NA	NA	0.11333032
rs10974789	9	4796032	G	A	0.167	-0.205	0.0119	1.49E-66	-0.084	0.0120	2.07E-12	NA	NA	0.10399221

Supplementary Table 4. The effect of low GMS-CF associated variants in a blood eQTL study consisting of 31,684 European individuals (Vosa et. al., 2018). Chr – Chromosome; SNP posi- SNP position (hg19 – GRCH37.p10); A1- effective allele; A2 – other allele; Freq – A1 allele frequency; b – Beta values; SE – Standard error; p – p-value.
